# Confirmation of neurometabolic diagnoses using age‐dependent cerebrospinal fluid metabolomic profiles

**DOI:** 10.1002/jimd.12253

**Published:** 2020-05-23

**Authors:** Tessa M. A. Peters, Udo F. H. Engelke, Siebolt de Boer, Ed van der Heeft, Cynthia Pritsch, Purva Kulkarni, Ron A. Wevers, Michèl A. A. P. Willemsen, Marcel M. Verbeek, Karlien L. M. Coene

**Affiliations:** ^1^ Department of Laboratory Medicine, Translational Metabolic Laboratory (TML) Radboud University Medical Center Nijmegen The Netherlands; ^2^ Department of Neurology, Donders Institute for Brain, Cognition and Behavior Radboud University Medical Center Nijmegen The Netherlands; ^3^ Department of Pediatric Neurology Donders Institute for Brain, Cognition and Behavior, Radboud University Medical Center Nijmegen The Netherlands

**Keywords:** biomarkers, CSF, mass spectrometry, metabolomics, neurometabolic disorders

## Abstract

Timely diagnosis is essential for patients with neurometabolic disorders to enable targeted treatment. Next‐Generation Metabolic Screening (NGMS) allows for simultaneous screening of multiple diseases and yields a holistic view of disturbed metabolic pathways. We applied this technique to define a cerebrospinal fluid (CSF) reference metabolome and validated our approach with patients with known neurometabolic disorders. Samples were measured using ultra‐high‐performance liquid chromatography‐quadrupole time‐of‐flight mass spectrometry followed by (un)targeted analysis. For the reference metabolome, CSF samples from patients with normal general chemistry results and no neurometabolic diagnosis were selected and grouped based on sex and age (0‐2/2‐5/5‐10/10‐15 years). We checked the levels of known biomarkers in CSF from seven patients with five different neurometabolic disorders to confirm the suitability of our method for diagnosis. Untargeted analysis of 87 control CSF samples yielded 8036 features for semiquantitative analysis. No sex differences were found, but 1782 features (22%) were different between age groups (*q* < 0.05). We identified 206 diagnostic metabolites in targeted analysis. In a subset of 20 high‐intensity metabolites and 10 biomarkers, 17 (57%) were age‐dependent. For each neurometabolic patient, ≥1 specific biomarker(s) could be identified in CSF, thus confirming the diagnosis. In two cases, age‐matching was essential for correct interpretation of the metabolomic profile. In conclusion, NGMS in CSF is a powerful tool in defining a diagnosis for neurometabolic disorders. Using our database with many (age‐dependent) features in CSF, our untargeted approach will facilitate biomarker discovery and further understanding of mechanisms of neurometabolic disorders.

**Synopsis**

Next‐Generation Metabolic Screening in CSF will facilitate biomarker discovery and further understanding of mechanisms of neurometabolic disorders, taking age‐dependency into account.

## 1 INTRODUCTION

Neurometabolic disorders are a group of inherited diseases that mainly affect the central nervous system. Even though the individual disorders are rare, collectively they represent an important category of severe but potentially treatable neurological disorders. The phenotypic manifestation of neurometabolic disorders can be very heterogeneous, for example, ranging from epilepsy to movement disorders to developmental delay. The common theme is presentation at a (very) young age. Coming to a correct diagnosis as soon as possible can be life saving for a child, as specific therapy can only be initiated once the metabolic defect is identified. Furthermore, understanding of the disturbed metabolic pathways of a disease may also lead to new opportunities for treatment.

Currently, in parallel to genetic diagnostics, neurometabolic disorders are functionally evaluated by both imaging techniques (magnetic resonance imaging and spectroscopy) and biochemical analyses of a limited set of known (metabolite) biomarkers in body fluids. Specific metabolic perturbations for neurometabolic diseases can often only be detected in cerebrospinal fluid (CSF). However, this strategy prevents the identification of novel disorders affecting unexplored metabolic pathways. Therefore, there is a need for a more holistic approach to biochemical diagnostics for neurometabolic disorders. We recently described such an approach, called Next‐Generation Metabolic Screening (NGMS), applied to inborn errors of metabolism (IEMs) using plasma samples.^1^ This approach comprises metabolomic analysis using high resolution ultra‐high‐performance liquid chromatography‐quadrupole time of flight mass spectrometry (UHPLC‐QTOF‐MS), allowing simultaneous measurement of hundreds of metabolites and diagnosis of more than 50 IEMs. By extending NGMS to CSF, we now aim to improve the diagnostic process for patients with neurometabolic diseases and open the way for the discovery of novel biomarkers and yet unknown metabolic diseases.

As UHPLC‐QTOF‐MS is a semiquantitative technique, the detection of deviating metabolites relies on comparison to controls. Biological and preanalytical variation within the control group may hamper such comparisons. Therefore, we present the metabolomic profile of control CSF samples obtained from children aged 0 to 15 years (the typical age of presentation for neurometabolic disorders), defining which metabolites can be identified using NGMS and the degree of variation in metabolite levels introduced by sex, age, and CSF fraction. Furthermore, we demonstrate the application of NGMS in CSF for the diagnosis of neurometabolic disorders, taking into account the variation of our control cohort.

## 2 MATERIALS AND METHODS

### 2.1 Study population and sample collection

Controls: leftover CSF samples (storage at −80°C) were selected based on the following criteria: (a) subject age 0 to 15 years old, (b) collected in 2016, 2017, or 2018, (c) erythrocyte and leukocyte count in CSF within reference range, (d) if available, routine CSF parameters hemoglobin, bilirubin, IgG production, glucose, lactate and protein within reference range, and (e) clinical data of the subject, as investigated by a pediatric neurologist, did not suggest the presence of a disease associated with abnormal CSF results. In accordance with local legislation, we only included samples of patients who did not reject to the anonymized use of their left‐over material for validation purposes. To ensure anonymity, all samples were labeled with a study number based on sex and age group (0‐2, 2‐5, 5‐10, 10‐15 years old).

To study potential concentration gradients of metabolites, CSF was used from a 6‐year‐old patient with increased intracranial pressure of unknown origin (despite extensive diagnostic work‐up). 20 mL of CSF was withdrawn and divided into four subsequent fractions of 5 mL before storage at −80°C.

Patient CSF samples were collected from the local clinical archive. Informed consent for the use of these samples for research purposes had been obtained previously. Diagnoses included isovaleric acidemia (IVA; OMIM #243500), succinic semialdehyde dehydrogenase (SSADH) deficiency (OMIM #271980), N‐acetylneuraminic acid synthase (NANS) deficiency (OMIM #610442), dihydropyrimidinase (DHP) deficiency (OMIM #222748), and aromatic l‐amino acid decarboxylase (AADC) deficiency (OMIM #608643). For all diagnoses except the latter, heparin plasma samples collected on the same date as the respective CSF sample were included as well. For each patient, the diagnosis was confirmed on both the genetic and the metabolic level. Moreover, the patients with NANS (patient 9)^2^ and DHP deficiency^3^ and one of the AADC deficiency patients (patient 2)^4^ have previously been described in detail.

### 2.2 Sample preparation and UHPLC‐QTOF‐MS analysis

CSF samples were analyzed by the reversed‐phase UHPLC‐QTOF‐MS method previously described in detail for heparin‐anticoagulated plasma,^1^ with some minor adjustments. For the positive ionization mode, an injection volume of 1.0 μL rather than 2.0 μL was used. All analytical batches included a quality control (QC) sample, a random patient pool (RPP) sample, a procedure blank (PB) and an internal standard solution. The QC sample was prepared by pooling 100 μL of the collected samples from the control cohort, while the RPP sample was made by pooling 30 leftover CSF samples from patients (age 0‐47 years old; 53% male). The pools were thoroughly mixed, centrifuged and 100 μL aliquots were stored at −80°C. All samples, except the QC, were measured in duplicate and in antiparallel order; the QC was measured after each 10 injections. The PB was a milli‐Q water sample prepared in the same way as the CSF samples.

### 2.3 Untargeted analysis of control cohort

For untargeted analysis, raw data acquired from the UHPLC‐QTOF‐MS runs were aligned using the R package “xcms” (XCMS version 3.4.4 running under R version 3.5.2; see Table S1 for used parameters).^5^ The extracted features (ie, the combination of an accurate mass‐to‐charge ratio (*m*/*z*), retention time (RT) and for each sample an intensity) were preprocessed by selecting those within an *m*/*z* range of 70 to 700, RT between 0.4 and 16 min, intensity in the PB lower than 10% of the mean intensity in the control cohort CSF samples, and with an intensity of ≥10 000 in at least one sample. For semiquantitative analyses, we averaged duplicate measurements. In the case of between‐run comparisons, we normalized feature intensities by dividing them by the average intensity in the pooled QC. Only features for which the coefficient of variation (CV) in the RPP sample was ≤20% after normalization were used in these between‐run comparisons.

### 2.4 Targeted analysis of IEM‐related metabolites

Annotation was performed using a predefined panel of 322 metabolites known to be associated with IEMs (see Table S2). Information on established RT of reference compounds, available for 265 metabolites (82%), was included in this IEM panel to allow for high confidence identification according to the guidelines of the Metabolomics Standards Initiative.^6^ Features were identified based on both *m*/*z* and RT of a reference compound (two orthogonal properties, classified as level 1), or *m*/*z* only (level 2). Features were matched to the IEM panel based on a <5 ppm deviation for mass accuracy and a <10% relative RT difference from the reference compound measurement (if available). Of note, only [M+H]^+^ and [M+Na]^+^ adducts for the positive mode and [M−H]^−^ and [M+Cl]^−^ adducts for the negative mode were included in this annotation. Initially, we matched features from the XCMS list obtained in untargeted analysis. In case a metabolite was not found, Agilent MassHunter Personal Compound Database and Library Manager Software version B.04.00, build 92.0 was used to further check its presence in the raw data. The 20 metabolites with the highest signal intensity as well as 10 metabolites that served as biomarkers for our patient samples (see section 2.5) were selected for semiquantitative analysis.

We studied the literature to identify metabolites with known concentration gradients in CSF prior to our study of the influence of the rostrocaudal gradient on metabolite levels. For each metabolite, matching features were identified in the four CSF fractions as described above.

### 2.5 Confirmation of neurometabolic diagnoses in CSF

We determined the performance of our NGMS method compared to the targeted amino acid and neurotransmitter assays that are currently used in diagnostics. To this end, we performed these targeted assays for 10 control samples from our cohort and one to three patient samples for six amino acids and four neurotransmitter metabolites. Amino acid analysis in CSF was performed using ion‐exchange chromatography on an amino acid analyzer (Biochrom 30, Biochrom, Cambourne, UK) according to the procedure recommended by the manufacturer. Neurotransmitter metabolites in CSF were investigated by high‐performance liquid chromatography with fluorimetric detection as previously described.^4^ The resulting quantitative concentrations were compared to the semiquantitative intensities derived from NGMS.

We then applied NGMS to samples of patients with a known neurometabolic diagnosis. Features that significantly differed from controls and could be annotated using the IEM panel were selected and checked for known biomarkers of the respective disease of the patient.

### 2.6 Statistical analysis

Sex‐related differences were tested by Wilcoxon signed‐rank test to all selected features, followed by Benjamini‐Hochberg correction with a false discovery rate (FDR) of 5%. Differences between age groups were tested by a Kruskal‐Wallis test with the same correction. For targeted analysis, we used the feature with the highest non‐saturated intensity for each metabolite for semiquantitative analysis. Of note, the Benjamini‐Hochberg correction is based on the number of comparisons within an analysis. Therefore, it is less stringent in targeted analysis (aimed at 30 features) than in untargeted analysis (aimed at thousands of features). *Z*‐scores were calculated to assess variation within the control group. For the analysis of CSF fractions and for comparison of NGMS to targeted assays, we calculated *R* ^2^ and the relative beta coefficient (ie, the beta coefficient divided by the mean; *β*_rel_) using linear regression. All statistics were performed using R version 3.5.2.

Features significantly different between a single patient and controls were determined by *t* tests followed by Bonferroni‐Holm correction as described previously.^1^ The fold change was calculated by dividing the mean intensity in the patient by the median intensity in the controls. In case of decreases, the variation between patient and controls is relatively small compared to the within‐group variation of the controls. Consequently, such features will not always reach our stringent significance threshold. Therefore, all annotated features with a fold change of ≤−2 were also screened for biomarkers known to be decreased in the respective disorder. For age‐dependent metabolites, the analysis was repeated using an age‐matched control group.

## 3 RESULTS

### 3.1 Untargeted analysis of control cohort

The demographics of the 87 selected control samples are specified in Table S3. We extracted 39 855 features after XCMS‐based feature detection and alignment (18 751 in positive mode, and 21 104 in negative mode). After preprocessing, 13 355 features remained. For further semiquantitative analysis, the intensities of the preprocessed features were normalized based on the QC sample. Those which exceeded a CV of 20% in the RPP sample after normalization were filtered out, leaving 8036 features. None of these were significantly different between males and females. 1782 features (22%) significantly differed between age groups (*q* < 0.05).

### 3.2 Targeted analysis of IEM‐related metabolites

**FIGURE 1:**
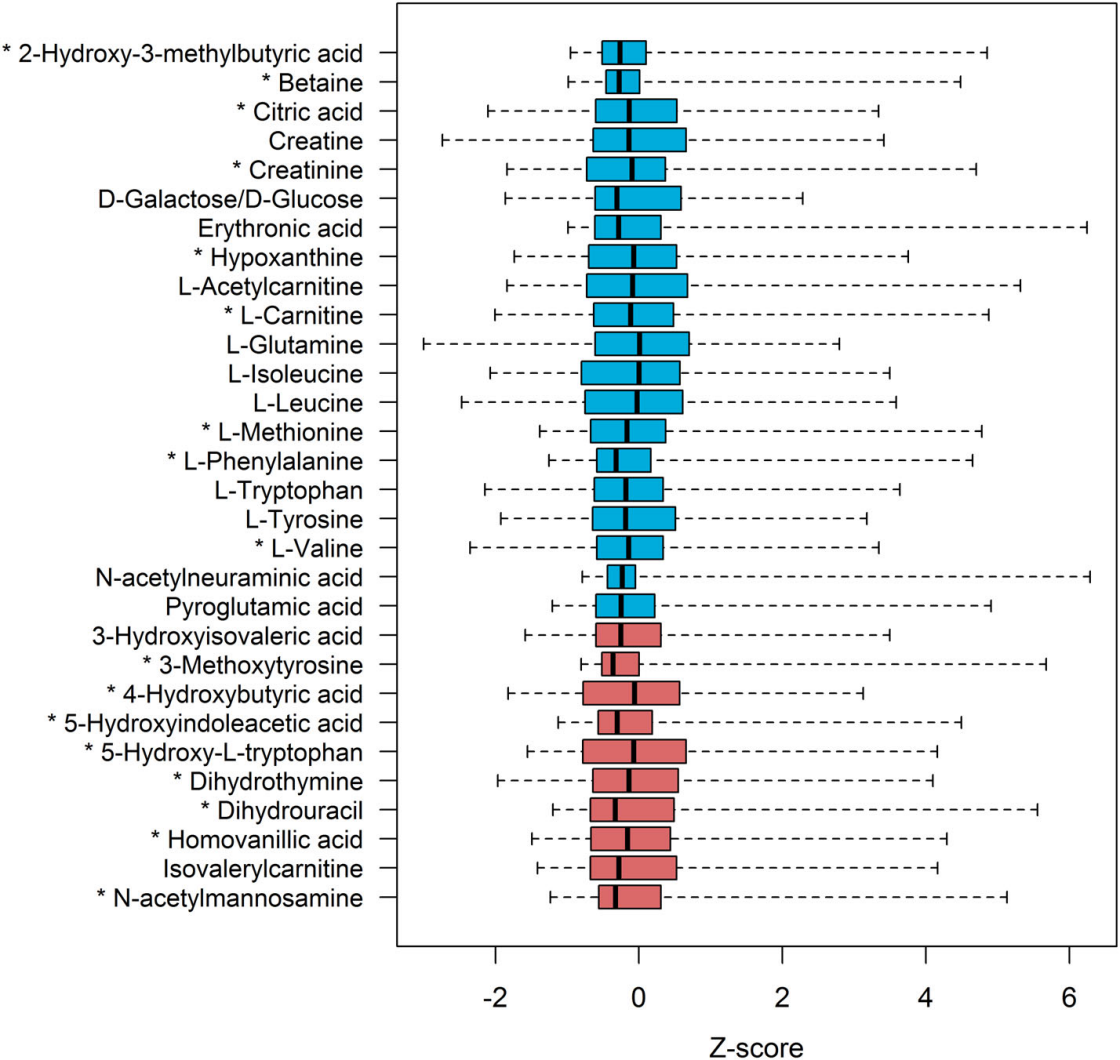
*Z*‐scores of the 20 metabolites with the highest median intensity (blue) and 10 metabolites that serve as biomarkers in this study (red) as measured in 87 control samples. Boxes show median and second and third quartiles. Whiskers extend to minimum and maximum values. Metabolites with an asterisk (*) are significantly different between age groups: see Figure S1 for details

Features extracted from the control cohort were matched against a predefined panel (Table S2). This resulted in detection of 206 metabolites, of which 176 with a level 1 identification. For a subset of 20 high intensity metabolites and 10 biomarkers (indicated in Table S2), semiquantitative analysis was performed. Despite less stringent multiple testing correction, there were still no metabolites with a significant difference based on sex (Table S4). However, 17 (57%) of these 30 metabolites were age‐dependent. Figure 1 provides an overview of the variation for these metabolites, represented by *Z*‐scores. Figure S1 shows the *Z*‐scores per age group together with the FDR‐corrected *P*‐values for each metabolite.

We identified 13 metabolites which were previously reported to exist in a rostrocaudal concentration gradient in CSF (Table 1), most of which are involved in neurotransmitter metabolism. For all metabolites except uric acid, an increased concentration in the higher fraction numbers was expected based on the literature, thus a positive slope (*β*_rel_). For 12 of the 13 metabolites, we found a corresponding feature in the fractions for which *R* ^2^ and *β*_rel_ could be calculated. Figure S2 shows scatter plots including the linear regression curve for each metabolite. Several metabolites showed the expected high correlation among the four fractions, especially 5‐hydroxyindoleacetic acid, homovanillic acid, and methylimidazoleacetic acid.

**TABLE 1:** Metabolites with concentration gradients in CSF as identified from literature

Metabolite	Reference	ID level	*R* ^2^	*β*_rel_
3,4‐Dihydroxyphenylacetic acid	^18^	2	0.90	0.071
5‐Hydroxyindoleacetic acid	^18^, ^19^, ^20^	1	0.97	0.109
5‐Hydroxytryptophol	^19^	2	0.36	−0.040
Creatinine	^22^	1	0.95	0.014
γ‐Aminobutyric acid	^23^	1	0.51	−0.054
Homocarnosine	^23^	1	0.48	0.069
Homovanillic acid	^18^, ^20^	1	0.96	0.125
Hypoxanthine	^22^	1	0.81	0.031
1‐Methylhistamine	^21^	0	NA	NA
Methylimidazoleacetic acid	^21^	2	0.93	0.188
Uric acid	^22^	1	0.60	−0.041
Vanylglycol	^20^	1	0.07	−0.009
Xanthine	^22^	1	0.81	0.043

*Note*: The coefficient of determination (*R* ^2^) and the relative beta coefficient (*β*_rel_) were calculated using linear regression. Identification (ID) levels: 1 = identification based on *m*/*z* and RT of a reference compound, 2 = putative identification based on *m*/*z* only, 0 = not identified. NA = not available.

### 3.3 Confirmation of neurometabolic diagnoses in CSF

We compared semiquantitative intensities from NGMS to quantitative concentrations from targeted assays for amino acids (n = 6) and neurotransmitter metabolites (n = 4) in 13 samples. This generally showed a good correlation (*R* ^2^ = 0.66‐1.00; see Figure S3).

Table 2 lists the findings from our samples of patients with a known neurometabolic diagnosis. For each disorder, at least one specific biomarker could be identified in CSF, confirming the diagnosis. Some biomarkers had a stronger deviation from controls in CSF than in plasma (3‐hydroxyisovaleric acid, N‐acetylmannosamine), while others had a lower fold change (isovalerylglycine).

**TABLE 2:** Identified biomarkers in samples of patients with a confirmed neurometabolic diagnosis

Diagnosis (OMIM)	n	Biomarker(s)	CSF	Plasma	Age‐dependent in CSF?^a^
Isovaleric acidemia (243500)	1	Isovalerylcarnitine	↑↑	↑↑	No (*q* = 0.52)
		Isovalerylglycine	↑^b^	↑↑↑	ND^d^
		3‐Hydroxyisovaleric acid	↓↓^c^	↓^c^	No (*q* = 0.89)
Succinic semialdehyde dehydrogenase deficiency (271980)	2	4‐Hydroxybutyric acid	↑↑↑/↑↑↑	↑↑↑/NA	Yes (*q* = 1.7E−04)
N‐acetylneuraminate synthase deficiency (610442)	1	N‐acetylmannosamine	↑↑	↑	Yes (*q* = 7.1E−03)
Dihydropyrimidinase deficiency (222748)	1	Dihydrothymine	↑↑	↑↑	Yes (*q* = 0.017)
		Dihydrouracil	↑	↑	Yes (*q* = 0.039)
		Thymine	↑↑↑	↑↑↑	ND^d^
		Uracil	↑^b^	↑	ND^d^
Aromatic l‐amino acid decarboxylase deficiency (608643)	2	3‐Methoxytyrosine	↑↑/↑^c^	NA/NA	Yes (*q* = 6.3E−09)
		5‐Hydroxytryptophan	↑^c^/↑	NA/NA	Yes (*q* = 6.7E−05)
		5‐Hydroxyindoleacetic acid	↓^c^/↓^c^	NA/NA	Yes (*q* = 2.5E−10)
		Homovanillic acid	↓^c^/↓↓^c^	NA/NA	Yes (*q* = 1.5E−07)

*Note*: Arrows indicate increased (↑) or decreased (↓) intensity of the indicated feature in the patient sample compared with controls. One arrow: fold change 1‐10, two arrows: fold change 10‐100, three arrows: fold change >100.

Abbreviations: n, number of patients; NA, not analyzed; ND, not determined.

^a^ Based on *q*‐values (=FDR‐adjusted *P*‐values) from the 30 IEM metabolite subanalysis.

^b^ Observed and abnormal in raw data but not observed in aligned data.

^c^ Not selected by standard statistical testing, corrected *P*‐value >.05.

^d^ Intensity in controls is too low for semiquantitative analysis.

**FIGURE 2:**
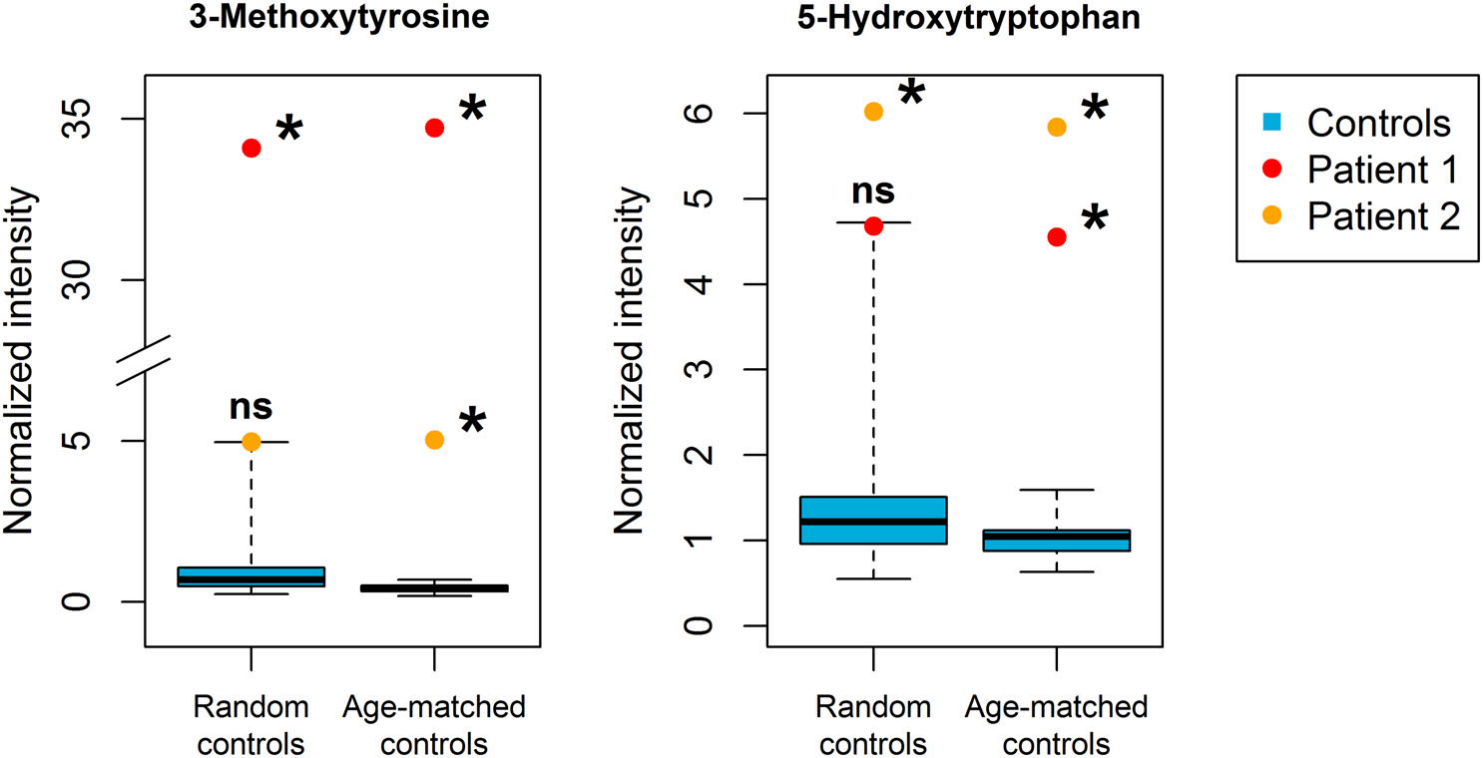
Analysis of 3‐methoxytyrosine and 5‐hydroxytryptophan in AADC deficiency patients compared to random and age‐matched controls. Boxplots (blue) represent data from control samples. Boxes show median and second and third quartiles of the control group. Whiskers extend to minimum and maximum values. Patient samples are individually plotted as points (red/orange). * = Bonferroni Holm‐corrected *P*‐value < .05 (when comparing the single patient to the controls), ns = not significant (*P* ≥ .05). The figure shows the relevance of working with age‐matched controls

An abnormally low abundance of a metabolite may not always be significant because the difference between patient and controls is relatively small when compared to variation within the control group. However, increases are expected to always reach the significance level of *P* < .05 (after correction). For two biomarkers, this was not the case: 3‐methoxytyrosine in one AADC deficiency patient, and 5‐hydroxytryptophan in the other. Since the concentrations of these metabolites were age‐dependent in our control cohort study, the analysis was repeated using an age‐matched control group. In the age‐matched reanalysis, concentrations of both 3‐methoxytyrosine and 5‐hydroxytryptophan were significantly increased in both patients (Figure 2).

## 4 DISCUSSION

NGMS analysis of 87 control CSF samples from children aged 0 to 15 years resulted in a reference metabolome consisting of 8036 features suitable for reproducible, semiquantitative analysis, including features representing 206 metabolites with a known connection to IEM. Significant differences were apparent between different age groups in both untargeted and targeted analysis. All neurometabolic diagnoses included in this study could be confirmed based on NGMS measurements in CSF samples of patients. Of note, for two patients (those with AADC deficiency), age‐matched controls were required to detect the complete expected biomarker pattern.

Using XCMS, we initially extracted a total of 39 855 features from our CSF measurements. From our experience, plasma measurements usually yield approximately 80 000 features. This difference suggests that plasma contains more metabolites than CSF. A possible explanation is that plasma contains more exogenous metabolites, for example, metabolites originating from food that do not reach the CSF. However, biomarker research is usually aimed at elucidating the endogenous metabolism. Thus, the use of CSF may be advantageous due to less interference by exogenous metabolites that are not of direct interest. Furthermore, analysis of CSF rather than plasma is already known to be essential for the biochemical diagnosis of some neurometabolic disorders, as is the case with AADC deficiency.^7^

Previously, an extensive list of metabolites identified by “untargeted” metabolomics in CSF of patients aged 0 to 20 years has been published.^8^ While the use of a large metabolite library is an advantage when annotating features, it also means that the analysis is no longer truly untargeted. Consequently, the discovery of unknown metabolites is no longer possible. This is also the case for several published targeted CSF metabolomics methods.^9^, ^10^ These can also accurately measure biomarkers for multiple neurometabolic diagnoses, including antiquitin deficiency and AADC deficiency. However, the main advantage of our method lies in obtaining a full unannotated dataset. This allows us to zoom in on any feature that may be divergent in a patient in the future and perform semiquantitative analyses as showcased for the IEM panel metabolites in this report. If the feature cannot be identified using our library or relevant databases (Human Metabolome Database [HMDB],^11^ Metlin^12^), additional techniques may be used to elucidate the molecular structure, for example nuclear magnetic resonance (NMR) or infrared (IR) ion spectroscopy.^2^, ^13^, ^14^ To subsequently make a newly identified metabolite biomarker available in a diagnostic setting, development of a targeted assay may be required to provide quantitative, high‐throughput results.

Another study recently also presented an untargeted metabolomics approach in CSF for diagnosis of (neuro)metabolic disorders.^15^ In this study, 1811 mass peaks were identified, but as signals with unknown identification were excluded from analysis, comparing this to our 8036 features is difficult. Furthermore, the methodology was based on direct‐infusion high‐resolution MS, without prior chromatographic separation. Thus, only the accurate mass was used to annotate metabolites, which was done by matching these to the HMDB. However, the human metabolome comprises many compounds with equal molecular mass that will not be distinguished in this way. Of note, even when using an LC system, we found a few pairs of isomers that had the same retention time and thus could still not be distinguished. Therefore, not only a second, but sometimes even a third orthogonal dimension of information (eg, an NMR or IR spectrum) is necessary for reliable feature identification.

As with all metabolomic approaches, we were not able to get a full coverage of the CSF metabolome. First, metabolites must be susceptible to ionization and be present in sufficiently high concentrations to be detected by QTOF‐MS. Furthermore, detection and (semi‐)quantification of polar metabolites is limited by the use of a reversed‐phase column. The latter is an obstacle in the assessment of sugars, which are generally highly polar and therefore only partially separated. Indeed, in our study, we were not able to discriminate between glucose and galactose, as they have the same retention time on the used column. Such problems may be improved by also performing measurements using a hydrophilic liquid interaction chromatography (HILIC) column,^16^ which will enhance the retention and separation of polar metabolites and thus increase the coverage.

Our results with regard to the effect of sex and age are in line with daily practice of our diagnostic laboratory: while age‐dependent reference ranges are applied to several targeted assays, sex is usually not taken into account. A previous metabolomic study did find sex differences in the concentrations of several metabolites, but this was in an elderly population.^17^ Possibly, effects of sex in metabolism are more pronounced after puberty. Based on the age‐dependent reference ranges, the used age range of 0 to 2 is relatively wide, as many metabolic changes occur during the first year of life. This choice is the consequence of a limited availability of samples within this range combined with the ethical restriction of using age groups rather than exact individual ages to prevent identification. However, we believe it would be valuable to study the 0 to 2 group in more detail. Future studies will therefore be aimed at collecting more CSF, allowing us to split the samples into narrower subgroups. Still, it should be noted that acquiring control CSF samples of this group is a challenge, not only in the present work, but also for any laboratory dealing with neurometabolic studies. After all, large series of controls are needed to establish a robust reference that adequately covers the remarkable changes for some metabolites during the first months or years of life.

When metabolites show a concentration gradient in CSF, the fraction that is used for measurement will influence the outcome. Therefore, we studied four consecutive 5 mL CSF fractions to confirm the gradient of a literature‐based selection of metabolites. While this 20 mL is only a small amount compared to the total CSF volume (estimated to be 150 mL in adults), larger amounts are not relevant for diagnostic purposes: if the gradient is too subtle to present in the first 20 mL, it will also not interfere with standard diagnostic testing. Moreover, it is not ethically justified to withdraw a larger volume because of the elevated risk of post‐dural‐puncture headaches. Several metabolites showed a high correlation among the four fractions, especially 5‐hydroxyindoleacetic acid, homovanillic acid, and methylimidazoleacetic acid. This fits with their presumed concentration gradients.^18^, ^19^, ^20^, ^21^ However, it is hard to draw definite conclusions with just a single patient and four samples. We will therefore continue our efforts to collect fractioned CSF to study concentration gradients in a larger number of cases. This will allow us to determine whether to take fraction into account when comparing a feature of interest between patient and control samples.

As demonstrated by data from the AADC deficiency patients, knowledge on the age‐dependency of metabolites can be vital for making the correct diagnosis. Conversely, deviating levels of an age‐dependent metabolite can be mistakenly interpreted as perturbations caused by the disease if age is not considered when comparing patients to controls. This may especially apply to very young patients (<0.5 years old), since several metabolites appear at much higher level at this age—also demonstrated by several metabolites with high levels in the 0 to 2 years age group in our study (Figure S1). Therefore, age‐matching should be incorporated in metabolomic analysis to prevent not only false negative, but also false positive results.

Our current study shows that NGMS in CSF is a powerful tool for diagnosis of neurometabolic disorders. We have established a reference CSF metabolome using a large, well‐characterized control CSF cohort. Based on this reference CSF metabolome, we were able to define age‐dependency of different metabolites. Our validation study indicates that information on age‐dependency can be crucial for the correct diagnosis of neurometabolic diseases. Now that we have proven the diagnostic power of NGMS in CSF, this methodology will allow us to extend this approach to yet unsolved cases with a suspicion of neurometabolic disease. Untargeted metabolomics will facilitate biomarker discovery and aid in further understanding of disease mechanisms in the brain.

## CONFLICT OF INTEREST

The authors declare that they have no conflict of interest.

## AUTHOR CONTRIBUTIONS

Tessa M. A. Peters designed and executed the experiments, performed data analysis and interpretation, and drafted the manuscript. Udo F. H. Engelke contributed to experimental design, data analysis and data interpretation, and revised the manuscript. Siebolt de Boer performed analytical measurements, was involved in data processing and revised the manuscript. Ed van der Heeft set up and validated the method, aided in analytical measurements, and revised the manuscript. Cynthia Pritsch selected the samples and revised the manuscript. Purva Kulkarni wrote the scripts used for data analysis, aided in data analysis, and revised the manuscript. Ron A. Wevers contributed to experimental design and revised the manuscript. Michèl A. A. P. Willemsen contributed to experimental design, selected the samples, and revised the manuscript. Marcel M. Verbeek contributed to experimental design, aided in data interpretation, and revised the manuscript. Karlien L. M. Coene contributed to experimental design, aided in data interpretation and initial drafting of the manuscript, and revised the manuscript.

## DATA AVAILABILITY STATEMENT

Data supporting the findings of this study are available as electronic supplementary material.

## ETHICS STATEMENT

All procedures followed were in accordance with the ethical standards of the responsible committee on human experimentation (institutional and national) and with the Helsinki Declaration of 1975, as revised in 2000. The study has been approved by the Commissie Mensgebonden Onderzoek Radboudumc under file number 2016‐3011 and titles “Metabolomic characterization of reference cerebrospinal fluid in children” (for control samples) and “Metabolomics in cerebrospinal fluid of patients with known and uncharacterized neurometabolic disorders” (for patient samples). All patients (or their guardians) approved of the possible use of their left‐over samples for method validation purposes, in agreement with institutional and national legislation. This article does not contain any studies with animal subjects performed by the any of the authors.

## Supporting information


**Appendix** **S1.** Supporting Information.
