# Supplementary material for: Confirmation of neurometabolic diagnoses using age‐dependent cerebrospinal fluid metabolomic profiles
Source: J Inherit Metab Dis. 2020 May 23;43(5):1112–20. doi: 10.1002/jimd.12253 (PMC7540372; doi:10.1002/jimd.12253)
Supplement: Supplementary file 1 — Appendix S1. Supporting Information. [file JIMD-43-1112-s001.pdf]

Supplementary material

Supplementary Table 1. Parameters used to convert the raw data to .mzData in Agilent Quantitative Analysis and to align the .mzData files using the "xcms" package in R.

Parameters for conversion to .mzData using Agilent MassHunter Qualitative Analysis B.07.00		
Settings	MS level	All
	MS storage	Centroid data (Compute deisotope: unchecked)
Peak Filters (MS)	Height filters	Absolute height >= 500 counts
	Maximum number of peaks	Limit (by height) to the largest: unchecked
Charge state (MS)	Isotope grouping	Peak spacing tolerance: 0.0025 *m/z*, plus 7.0 ppm<br>Isotope model: Common organic molecules
	Charge state	Limit assigned states to a maximum of: 1<br>Treat ions with unassigned charge as singly-charge: unchecked
Parameters for alignment using XCMS 3.4.4 running under R version 3.5.2		
xcmsSet	Method	centWave
	Ppm	15
	Peakwidth	5-10
	Snthresh	10
	Mzdiff	0.01
	Integrate	2
	Prefilter	3-500
	Noise	0
retcor	Method	obiwarp
	profStep	1
Group	Bw	3
	Minfrac	0.1
	Mzwid	0.1
	Minsamp	1
	Max	1000

Supplementary Table 2. Full list of the 322 metabolites from the Inborn Errors of Metabolism (IEM) panel and whether they are present in the control CSF (n=87) or not.

Metabolite	HMDB ID	Formula	Monoisotopic mass	Detected in CSF	ID level[a]	Remark
(R)-3-Hydroxyisobutyric acid	HMDB0000336	*C4H8O3*	104.0473	+	1	
(S)-β-Aminoisobutyric acid	HMDB0002166	*C4H9NO2*	103.0633	+	1	
2,3-Dihydroxy-2-methylbutanoic acid	HMDB0029576	C5H10O4	134.0579	+	2	
2-Hydroxy-3-methylbutyric acid	HMDB0000407	*C5H10O3*	118.0630	+	1	Top 20 highest intensity
2-Hydroxy-3-methylpentanoic acid	HMDB0000317	*C6H12O3*	132.0786	+	1	
2-Hydroxyadipic acid/<br>3-Hydroxymethylglutaric acid	HMDB0000321/<br>HMDB0000355	*C6H10O5*	162.0528	+	1	
2-Hydroxybutyric acid	HMDB0000008	*C4H8O3*	104.0473	+	1	
2-Hydroxycaproic acid	HMDB0001624	*C6H12O3*	132.0786	+	1	
2-Hydroxydecanedioic acid/<br>3-Hydroxysebacic acid	HMDB0000424/<br>HMDB0000350	C10H18O5	218.1154	+	2	
2-Methyl-3-hydroxybutyric acid	HMDB0000354	*C5H10O3*	118.0630	+	1[b]	
2-Methyl-3-ketovaleric acid/<br>Mevalonolactone	HMDB0000408/<br>HMDB0006024	*C6H10O3*	130.0630	+	2	
2-Methylacetoacetic acid	HMDB0003771	*C5H8O3*	116.0473	+	2	
2-Methylbutyroylcarnitine	HMDB0000378	*C12H23NO4*	245.1627	+	1	
2-Methylglutaric acid/<br>Solerol	HMDB0000422/<br>HMDB0002173	*C6H10O4*	146.0579	+	2	
2-Oxovaleric acid	HMDB0001865	*C5H8O3*	116.0473	+	1	
3a,4b,7a-Trihydroxy-5b-cholanoic acid	HMDB0000320	C24H40O5	408.2876	+	2	
3-Hydroxyadipic acid	HMDB0000345	*C6H10O5*	162.0528	+	2	
3-Hydroxybutyric acid	HMDB0000357	*C4H8O3*	104.0473	+	1	
3-Hydroxyhexanoic acid	HMDB0061652	*C6H12O3*	132.0786	+	2	

3-Hydroxyisovaleric acid	HMDB0000754	*C5H10O3*	118.0630	+	1	Biomarker in this study (IVA)
3-Hydroxyisovalerylcarnitine	HMDB0061189	C12H23O5N1	262.1654	+	1[b]	
3-Methoxytyrosine	HMDB0001434	C10H13NO4	211.0845	+	1	Biomarker in this study (AADC deficiency)
3-Methyl-2-oxovaleric acid	HMDB0000491	*C6H10O3*	130.0630	+	1	
3-Methylglutaconic acid	HMDB0000522	*C6H8O4*	144.0423	+	1	
3-Methylglutarylcarnitine	HMDB0000552	*C13H23NO6*	289.1525	+	1	
4-Hydroxybutyric acid	HMDB0000710	*C4H8O3*	104.0473	+	1	Biomarker in this study (SSADH deficiency)
4-Hydroxyisovaleric acid	HMDB0002011	*C5H10O3*	118.0630	+	2	
4-Hydroxyproline	HMDB0000725	*C5H9NO3*	131.0582	+	1	
4-Pyridoxic acid	HMDB0000017	C8H9NO4	183.0532	+	1	
5-Hydroxyhexanoic acid	HMDB0000525	*C6H12O3*	132.0786	+	1	
5-Hydroxyindoleacetic acid	HMDB0000763	C10H9NO3	191.0582	+	1	Biomarker in this study (AADC deficiency)
5-Hydroxyisourate	HMDB0030097	C5H4N4O4	184.0233	+	1[b]	
5-Hydroxy-L-tryptophan	HMDB0000472	C11H12N2O3	220.0848	+	1	Biomarker in this study (AADC deficiency)
5-Hydroxytryptophol	HMDB0001855	C10H11NO2	177.0790	+	2	
5-Hydroxylysine	HMDB0000450	C6H14N2O3	162.1004	+	1	
6-Oxopiperidine-2-carboxylic acid	HMDB0061705	C6H9NO3	143.0582	+	1[b]	
7-Hydroxyoctanoic acid	HMDB0000486	C8H16O3	160.1099	+	2	
Acetylglycine	HMDB0000532	C4H7NO3	117.0426	+	1[b]	
Adenine	HMDB0000034	C5H5N5	135.0545	+	1	
Adenosine	HMDB0000050	*C10H13N5O4*	267.0968	+	1	
AICA-riboside	HMDB0062179	C9H14N4O5	258.0964	+	2	
Alpha-ketoisovaleric acid	HMDB0000019	*C5H8O3*	116.0473	+	1	
Aminoadipic acid	HMDB0000510	*C6H11NO4*	161.0688	+	1	

Arabinose	HMDB0029942	C5H10O5	150.0528	+	1	
Aspartic acid	HMDB0000191	C4H7NO4	133.0375	+	1	
Azelaic acid	HMDB0000784	C9H16O4	188.1049	+	2	
Beta-1,4-mannose-N-acetylglucosamine	HMDB0006535	C14H25NO11	383.1428	+	2	
Betaine	HMDB0000043	*C5H11NO2*	117.0790	+	1	Top 20 highest intensity
Biopterin	HMDB0000468	C9H11N5O3	237.0862	+	1	
Butanone	HMDB0000474	C4H8O	72.0575	+	2	
Butyrylcarnitine	HMDB0002013	*C11H21NO4*	231.1471	+	1	
Caproic acid	HMDB0000535	C6H12O2	116.0837	+	2	
Citric acid	HMDB0000094	C6H8O7	192.0270	+	1	Top 20 highest intensity
Citrulline	HMDB0000904	C6H13N3O3	175.0957	+	1	
Creatine	HMDB0000064	C4H9N3O2	131.0695	+	1[b]	Top 20 highest intensity
Creatinine	HMDB0000562	C4H7N3O	113.0589	+	1[b]	Top 20 highest intensity
Cytidine	HMDB0000089	C9H13N3O5	243.0855	+	1	
Cytosine	HMDB0000630	C4H5N3O	111.0433	+	1	
Deoxycytidine	HMDB0000014	C9H13N3O4	227.0906	+	1	
Deoxyguanosine	HMDB0000085	*C10H13N5O4*	267.0968	+	1	
Deoxyinosine	HMDB0000071	C10H12N4O4	252.0859	+	1	
Deoxyuridine	HMDB0000012	C9H12N2O5	228.0746	+	1	
D-Galactose/<br>D-Glucose	HMDB0000143/<br>HMDB0000122	*C6H12O6*	180.0634	+	1	Top 20 highest intensity
Dihydrothymine	HMDB0000079	C5H8N2O2	128.0586	+	1	Biomarker in this study (DHP deficiency)
Dihydrouracil	HMDB0000076	C4H6N2O2	114.0429	+	1	Biomarker in this study (DHP deficiency)
Dimethyl sulfoxide	HMDB0002151	C2H6OS	78.0139	+	1	

Dimethylglycine/<br>Gamma-Aminobutyric acid	HMDB0000092/<br>HMDB0000112	*C4H9NO2*	103.0633	+	1	
Erythronic acid	HMDB0000613	C4H8O5	136.0372	+	1	Top 20 highest intensity
Ethylmalonic acid/<br>Glutaric acid	HMDB0000622/<br>HMDB0000661	*C5H8O4*	132.0423	+	1	
Fumaric acid	HMDB0000134	C4H4O4	116.0110	+	1	
Galactitol/<br>Mannitol	HMDB0000107/<br>HMDB0000765	C6H14O6	182.0790	+	1	
Gamma-Butyrolactone	HMDB0000549	C4H6O2	86.0368	+	2	
Glutaconic acid	HMDB0000620	*C5H6O4*	130.0266	+	1	
Glutamylphenylalanine	HMDB0000594	C14H18N2O5	294.1216	+	1	
Glutarylcarnitine	HMDB0013130	C12H21NO6	275.1369	+	1[b]	
Glycine	HMDB0000123	C2H5NO2	75.0320	+	1	
Glycolic acid	HMDB0000115	C2H4O3	76.0160	+	1	
Guanidoacetic acid	HMDB0000128	C3H7N3O2	117.0538	+	1[b]	
Guanosine	HMDB0000133	*C10H13N5O5*	283.0917	+	1	
Homocarnosine	HMDB0000745	C10H16N4O3	240.1222	+	1	
Homocitrulline	HMDB0000679	C7H15N3O3	189.1113	+	1	
Homocysteine	HMDB0000742	C4H9NO2S	135.0354	+	1	
Homovanillic acid	HMDB0000118	C9H10O4	182.0579	+	1	Biomarker in this study<br>(AADC deficiency)
Hydantoin-5-propionic acid	HMDB0001212	C6H8N2O4	172.0484	+	1	
Hydroxyisocaproic acid	HMDB0000746	*C6H12O3*	132.0786	+	1	
Hydroxykynurenine	HMDB0000732	C10H12N2O4	224.0797	+	1	
Hypoxanthine	HMDB0000157	*C5H4N4O*	136.0385	+	1	Top 20 highest intensity
Inosine	HMDB0000195	C10H12N4O5	268.0808	+	1	
Isobutyric acid	HMDB0001873	C4H8O2	88.0524	+	2	
Isobutyrylglycine	HMDB0000730	*C6H11NO3*	145.0739	+	1	

Isobutyryl-L-carnitine	HMDB0000736	*C11H21NO4*	231.1471	+	2	
Isovalerylcarnitine	HMDB0000688	*C12H23NO4*	245.1627	+	1	Biomarker in this study (IVA)
Itaconic acid	HMDB0002092	*C5H6O4*	130.0266	+	1	
Ketoleucine	HMDB0000695	*C6H10O3*	130.0630	+	1	
Kynurenic acid	HMDB0000715	C10H7NO3	189.0426	+	1	
L-2-Hydroxyglutaric acid	HMDB0000694	*C5H8O5*	148.0372	+	1	
L-Acetylcarnitine	HMDB0000201	*C9H17NO4*	203.1158	+	1	Top 20 highest intensity
L-Alanine/<br>Sarcosine	HMDB0000161/<br>HMDB0000271	*C3H7NO2*	89.0477	+	1	
L-Alloisoleucine	HMDB0000557	*C6H13NO2*	131.0946	+	1	
L-Arabitol/<br>Ribitol	HMDB0001851/<br>HMDB0000508	C5H12O5	152.0685	+	1	
L-Arginine	HMDB0000517	C6H14N4O2	174.1117	+	1	
L-Asparagine	HMDB0000168	*C4H8N2O3*	132.0535	+	1	
L-Carnitine	HMDB0000062	C7H15NO3	161.1052	+	1	Top 20 highest intensity
L-Dopa	HMDB0000181	C9H11NO4	197.0688	+	1	
L-Glutamic acid	HMDB0000148	*C5H9NO4*	147.0532	+	1	
L-Glutamine	HMDB0000641	*C5H10N2O3*	146.0691	+	1	Top 20 highest intensity
L-Glyceric acid	HMDB0006372	C3H6O4	106.0266	+	1	
L-Histidine	HMDB0000177	C6H9N3O2	155.0695	+	1	
L-Isoleucine	HMDB0000172	*C6H13NO2*	131.0946	+	1	Top 20 highest intensity
L-Kynurenine	HMDB0000684	C10H12N2O3	208.0848	+	2	
L-Lactic acid	HMDB0000190	*C3H6O3*	90.0317	+	1	
L-Leucine	HMDB0000687	*C6H13NO2*	131.0946	+	1	Top 20 highest intensity
L-Lysine	HMDB0000182	C6H14N2O2	146.1055	+	1	
L-Methionine	HMDB0000696	C5H11NO2S	149.0510	+	1	Top 20 highest intensity
L-Phenylalanine	HMDB0000159	C9H11NO2	165.0790	+	1	Top 20 highest intensity
L-Pipecolic acid	HMDB0000716	C6H11NO2	129.0790	+	1	

L-Proline	HMDB0000162	C5H9NO2	115.0633	+	1	
L-Serine	HMDB0000187	C3H7NO3	105.0426	+	1	
L-Threonine	HMDB0000167	C4H9NO3	119.0582	+	1	
L-Tryptophan	HMDB0000929	C11H12N2O2	204.0899	+	1	Top 20 highest intensity
L-Tyrosine	HMDB0000158	C9H11NO3	181.0739	+	1	Top 20 highest intensity
L-Valine	HMDB0000883	*C5H11NO2*	117.0790	+	1	Top 20 highest intensity
Maltotetraose	HMDB0001296	C24H42O21	666.2219	+	2	
Mesaconic acid	HMDB0000749	*C5H6O4*	130.0266	+	1	
Methionine sulfoxide	HMDB0002005	C5H11NO3S	165.0460	+	1	
Methylmalonic acid	HMDB0000202	*C4H6O4*	118.0266	+	1	
Methylsuccinic acid	HMDB0001844	*C5H8O4*	132.0423	+	1	
Mevalonic acid	HMDB0000227	C6H12O4	148.0736	+	1	
N1-Methyl-2-pyridone-5-carboxamide	HMDB0004193	*C7H8N2O2*	152.0586	+	1[b]	
N1-Methyl-4-pyridone-3-carboxamide	HMDB0004194	*C7H8N2O2*	152.0586	+	1[b]	
N2-gamma-Glutamylglutamine	HMDB0011738	C10H17N3O6	275.1117	+	1	
N6-Acetyl-L-lysine	HMDB0000206	*C8H16N2O3*	188.1161	+	1	
N8-Acetylspermidine	HMDB0002189	C9H21N3O	187.1685	+	2	
N-Acetylasparagine	HMDB0006028	*C6H10N2O4*	174.0641	+	1	
N-Acetylaspartylglutamic acid	HMDB0001067	C11H16N2O8	304.0907	+	1	
N-Acetylglutamic acid	HMDB0001138	C7H11NO5	189.0637	+	1	
N-Acetylglutamine	HMDB0006029	C7H12N2O4	188.0797	+	1	
N-Acetylhistidine	HMDB0032055	C8H11N3O3	197.0800	+	1	
N-Acetylisoleucine	HMDB0061684	*C8H15NO3*	173.1052	+	1[b]	
N-Acetyl-L-alanine	HMDB0000766	*C5H9NO3*	131.0582	+	1	
N-Acetyl-L-aspartic acid	HMDB0000812	C6H9NO5	175.0481	+	1	
N-Acetylleucine	HMDB0011756	*C8H15NO3*	173.1052	+	1	
N-Acetyl-L-methionine	HMDB0011745	C7H13NO3S	191.0616	+	1	

N-Acetyl-L-phenylalanine	HMDB0000512	*C11H13NO3*	207.0895	+	1	
N-Acetyl-L-tyrosine	HMDB0000866	C11H13NO4	223.0845	+	1	
N-Acetylmannosamine	HMDB0001129	C8H15NO6	221.0899	+	1	Biomarker in this study (NANS deficiency)
N-Acetylneuraminic acid	HMDB0000230	C11H19NO9	309.1060	+	1	Top 20 highest intensity
N-Acetylserine	HMDB0002931	*C5H9NO4*	147.0532	+	1[b]	
N-Acetylthreonine	HMDB0062557	*C6H11NO4*	161.0688	+	1	
N-Acetylvaline	HMDB0011757	*C7H13NO3*	159.0895	+	1	
N-Alpha-acetyllysine	HMDB0000446	*C8H16N2O3*	188.1161	+	1	
Neopterin	HMDB0000845	C9H11N5O4	253.0811	+	1	
Nicotinamide N-oxide	HMDB0002730	*C6H6N2O2*	138.0429	+	2	
N-lactoyl-(iso)leucine	HMDB0062176	*C9H17NO4*	203.1158	+	2	
N-lactoyl-Valine	HMDB0062181	C8H15NO4	189.1001	+	2	
N-Methylnicotinamide	HMDB0003152	C7H8N2O	136.0637	+	1	
N-α-Acetylcitrulline	HMDB0000856	C8H15N3O4	217.1063	+	1	
O-Adipoylcarnitine	HMDB0061677	*C13H23NO6*	289.1525	+	2	
Ornithine	HMDB0000214	C5H12N2O2	132.0899	+	1	
Orotic acid	HMDB0000226	C5H4N2O4	156.0171	+	1	
Orotidine	HMDB0000788	C10H12N2O8	288.0594	+	1	
Ortho-Hydroxyphenylacetic acid	HMDB0000669	*C8H8O3*	152.0473	+	1	
Oxoglutaric acid	HMDB0000208	C5H6O5	146.0215	+	1	
Pantothenic acid	HMDB0000210	C9H17NO5	219.1107	+	1	
Phenylacetic acid	HMDB0000209	C8H8O2	136.0524	+	1	
Phenyllactic acid	HMDB0000779	C9H10O3	166.0630	+	1	
Pimelic acid	HMDB0000857	*C7H12O4*	160.0736	+	1	
Pivaloylcarnitine	HMDB0041993	*C12H23NO4*	245.1627	+	1	
Propionylcarnitine	HMDB0000824	C10H19NO4	217.1314	+	1	
Pseudouridine	HMDB0000767	*C9H12N2O6*	244.0695	+	1	

Pyridoxal	HMDB0001545	C8H9NO3	167.0582	+	1	
Pyroglutamic acid	HMDB0000267	C5H7NO3	129.0426	+	1	Top 20 highest intensity
Pyruvic acid	HMDB0000243	C3H4O3	88.0160	+	1	
Saccharopine	HMDB0000279	C11H20N2O6	276.1321	+	1	
Sedoheptulose	HMDB0003219	C7H14O7	210.0740	+	1[b]	
Succinic acid	HMDB0000254	*C4H6O4*	118.0266	+	1	
Succinyladenosine	HMDB0000912	C14H17N5O8	383.1077	+	1[b]	
Succinylcarnitine	HMDB0061717	*C11H19NO6*	261.1212	+	2	
Taurine	HMDB0000251	C2H7NO3S	125.0147	+	1	
Thymidine	HMDB0000273	C10H14N2O5	242.0903	+	1	
Thymine	HMDB0000262	C5H6N2O2	126.0429	+	1	
Tiglic acid	HMDB0001470	C5H8O2	100.0524	+	2	
Tiglylcarnitine	HMDB0002366	C12H21NO4	243.1471	+	1[b]	
Trimethylamine N-oxide	HMDB0000925	C3H9NO	75.0684	+	1	
Uracil	HMDB0000300	C4H4N2O2	112.0273	+	1	
Ureidoisobutyric acid	HMDB0002031	*C5H10N2O3*	146.0691	+	1[b]	
Ureidopropionic acid	HMDB0000026	*C4H8N2O3*	132.0535	+	1	
Uric acid	HMDB0000289	C5H4N4O3	168.0283	+	1	
Uridine	HMDB0000296	*C9H12N2O6*	244.0695	+	1	
Urocanic acid	HMDB0000301	*C6H6N2O2*	138.0429	+	1	
Vanillactic acid	HMDB0000913	C10H12O5	212.0685	+	1	
Vanillic acid	HMDB0000484	*C8H8O4*	168.0423	+	1	
Vanylglycol	HMDB0001490	C9H12O4	184.0736	+	1	
Xanthine	HMDB0000292	*C5H4N4O2*	152.0334	+	1	
Xanthosine	HMDB0000299	C10H12N4O6	284.0757	+	1	
Xanthurenic acid	HMDB0000881	C10H7NO4	205.0375	+	1	
(E)-2-Methylglutaconic acid	HMDB0002266	*C6H8O4*	144.0423	-	-1	

(S)-2,3,4,5-Tetrahydropiperidine-2-carboxylate	HMDB0012130	C6H9NO2	127.0633	-	-1	
(S)-2-Methylbutanoic acid	HMDB0033742	*C5H10O2*	102.0681	-	-2	
11Z-Octadecenylcarnitine	HMDB0013338	C25H47NO4	425.3505	-	-2	
1-Pyrroline-2-carboxylic acid	HMDB0006875	C5H7NO2	113.0477	-	-1	
2,8-Dihydroxyadenine	HMDB0000401	C5H5N5O2	167.0443	-	-1	
2-Ethylhydracrylic acid	HMDB0000396	*C5H10O3*	118.0630	-	-2	
2-Methyl-3-hydroxyvaleric acid	HMDB0029166	*C6H12O3*	132.0786	-	-1	
2-Methylbutyrylglycine	HMDB0000339	*C7H13NO3*	159.0895	-	-1	
2-Methylcitric acid	HMDB0000379	C7H10O7	206.0427	-	-1	
2-Phenylpropionate	HMDB0011743	C9H10O2	150.0681	-	-2	
2-Pyrroloylglycine	HMDB0059778	C7H8N2O3	168.0535	-	-1	
2-trans,4-cis-Decadienoylcarnitine	HMDB0013325	C17H29NO4	311.2097	-	-1	
3, 5-Tetradecadiencarnitine	HMDB0013331	C21H37NO4	367.2723	-	-1	
3,4-Dihydroxybenzeneacetic acid	HMDB0001336	*C8H8O4*	168.0423	-	-1	
3,4-Dihydroxybenzeneacetic acid	HMDB0001336	*C8H8O4*	168.0423	-	-1	
3a,7a,12a-Trihydroxy-5b-cholestanoic acid	HMDB0000601	C27H46O5	450.3345	-	-1	
3-Hydroxyglutaric acid	HMDB0000428	*C5H8O5*	148.0372	-	-1	
3-Hydroxysuberic acid	HMDB0000325	C8H14O5	190.0841	-	-2	
3-Methyladipic acid	HMDB0000555	*C7H12O4*	160.0736	-	-1	
3-Methylcrotonylglycine	HMDB0000459	*C7H11NO3*	157.0739	-	-1	
3-Methylpimelic acid	HMDB0059757	*C8H14O4*	174.0892	-	-2	
3-Oxoadipic acid	HMDB0000398	*C6H8O5*	160.0372	-	-1	
3-Phenylpropionylglycine	HMDB0002042	*C11H13NO3*	207.0895	-	-1	
4-Hydroxy-L-glutamic acid	HMDB0002273	C5H7NO5	163.0481	-	-2	
4-Hydroxyphenylpyruvic acid	HMDB0000707	C9H8O4	180.0423	-	-1	
5a-Cholestane-3a,7a,12a,25-tetrol	HMDB0000520	C27H48O4	436.3553	-	-1	

5alpha-Cholestanol	HMDB0000908	C27H48O	388.3705	-	-2	
5-Aminolevulinic acid	HMDB0001149	*C5H9NO3*	131.0582	-	-1	
5b-Cholestane-3a,7a,12a,23S,25-pentol	HMDB0000483	C27H48O5	452.3502	-	-1	
5-Hydroxymethyluracil	HMDB0000469	C5H6N2O3	142.0378	-	-1	
7-Dehydrocholesterol	HMDB0000032	*C27H44O*	384.3392	-	-2	
8-Dehydrocholesterol	HMDB0002027	*C27H44O*	384.3392	-	-2	
8-Hydroxy-deoxyguanosine	HMDB0003333	*C10H13N5O5*	283.0917	-	-1	
9,12-Hexadecadienoylcarnitine	HMDB0013334	C23H41NO4	395.3036	-	-2	
9-Decenoylcarnitine	HMDB0013205	C17H31NO4	313.2253	-	-1	
9-Hexadecenoylcarnitine	HMDB0013207	C23H43NO4	397.3192	-	-2	
Acetoacetic acid	HMDB0000060	*C4H6O3*	102.0317	-	-1	
Adipic acid	HMDB0000448	*C6H10O4*	146.0579	-	-1	
Alanyl-Proline	HMDB0028695	C8H14N2O3	186.1004	-	-1	
Allopurinol	HMDB0014581	*C5H4N4O*	136.0385	-	-1	
Allysine	HMDB0001263	*C6H11NO3*	145.0739	-	-1	
Argininosuccinic acid	HMDB0000052	C10H18N4O6	290.1226	-	-1	
Aspartylglycosamine	HMDB0000489	C12H21N3O8	335.1329	-	-2	
Beta-Alanine	HMDB0000056	*C3H7NO2*	89.0477	-	-1	
Beta-Sitosterol	HMDB0000852	C29H50O	414.3862	-	-1	
Capryloylglycine	HMDB0000832	C10H19NO3	201.1365	-	-1	
Carnosine	HMDB0000033	C9H14N4O3	226.1066	-	-1	
Cholestane-3,7,12,25-tetrol-3-glucuronide	HMDB0010355	C33H56O10	612.3873	-	-1	
cis-4-Hydroxycyclohexylacetic acid	HMDB0000451	C8H14O3	158.0943	-	-2	
cis-5-Tetradecenoylcarnitine	HMDB0002014	C21H39NO4	369.2879	-	-1	
Cyclic pyranopterin monophosphate	HMDB0059639	C10H14N5O8P	363.0580	-	-2	
Cysteine-S-sulfate	HMDB0000731	C3H7NO5S2	200.9766	-	-2	
Decanoylcarnitine	HMDB0000651	C17H33NO4	315.2410	-	-1	

Deoxyadenosine	HMDB0000101	C10H13N5O3	251.1018	-	-1	
Dimethyl sulfone	HMDB0004983	C2H6O2S	94.0089	-	-1	
Dodecanoylcarnitine	HMDB0002250	C19H37NO4	343.2723	-	-2	
Dopamine	HMDB0000073	C8H11NO2	153.0790	-	-1	
Epinephrine	HMDB0000068	C9H13NO3	183.0895	-	-1	
epsilon-Caprolactone	HMDB0060476	C6H10O2	114.0681	-	-2	
Formiminoglutamic acid	HMDB0000854	*C6H10N2O4*	174.0641	-	-1	
Glycerol	HMDB0000131	C3H8O3	92.0473	-	-1	
Glycylproline	HMDB0000721	C7H12N2O3	172.0848	-	-1	
Guanine	HMDB0000132	C5H5N5O	151.0494	-	-1	
Hawkinsin	HMDB0002354	C11H17NO6S	291.0777	-	-2	
Hexanoylglycine	HMDB0000701	*C8H15NO3*	173.1052	-	-1	
Homogentisic acid	HMDB0000130	*C8H8O4*	168.0423	-	-1	
Hydroxypropionic acid	HMDB0000700	*C3H6O3*	90.0317	-	-1	
Isovaleric acid	HMDB0000718	*C5H10O2*	102.0681	-	-2	
Isovalerylglycine	HMDB0000678	*C7H13NO3*	159.0895	-	-1	
L-Cystathionine	HMDB0000099	C7H14N2O4S	222.0674	-	-1	
L-Cystine	HMDB0000192	C6H12N2O4S2	240.0238	-	-1	
L-Dihydroorotic acid	HMDB0003349	C5H6N2O4	158.0328	-	-1	
Leucyl-Proline	HMDB0011175	C11H20N2O3	228.1474	-	-1	
L-Hexanoylcarnitine	HMDB0000756	C13H25NO4	259.1784	-	-1	
L-Homocystine	HMDB0000676	C8H16N2O4S2	268.0551	-	-1	
Linoleyl carnitine	HMDB0006469	C25H45NO4	423.3349	-	-2	
L-Octanoylcarnitine	HMDB0000791	C15H29NO4	287.2097	-	-1	
L-Palmitoylcarnitine	HMDB0000222	C23H45NO4	399.3349	-	-1	
L-phenylalanyl-L-proline	HMDB0011177	C14H18N2O3	262.1317	-	-1	
Malic acid	HMDB0000744	C4H6O5	134.0215	-	-1	

Malonic acid	HMDB0000691	C3H4O4	104.0110	-	-1	
Malonylcarnitine	HMDB0002095	C10H17NO6	247.1056	-	-2	
Methylglutaric acid	HMDB0000752	*C6H10O4*	146.0579	-	-1	
Methylmalonylcarnitine	HMDB0013133	*C11H19NO6*	261.1212	-	-1	
N-Acetylproline	HMDB0094701	*C7H11NO3*	157.0739	-	-1	
N-Butyrylglycine	HMDB0000808	*C6H11NO3*	145.0739	-	-1	
N-Lactoyl-phenylalanine	HMDB0062175	C12H15NO4	237.1001	-	-1	
Norepinephrine	HMDB0000216	C8H11NO3	169.0739	-	-1	
O-Phosphoethanolamine	HMDB0000224	C2H8NO4P	141.0191	-	-1	
Oxalic acid	HMDB0002329	C2H2O4	89.9953	-	-1	
Oxoadipic acid	HMDB0000225	*C6H8O5*	160.0372	-	-1	
Oxypurinol	HMDB0000786	*C5H4N4O2*	152.0334	-	-1	
p-Cresol sulfate	HMDB0011635	C7H8O4S	188.0143	-	-1	
Phenylglyoxylic acid	HMDB0001587	C8H6O3	150.0317	-	-2	
p-Hydroxyphenylacetic acid	HMDB0000020	*C8H8O3*	152.0473	-	-1	
Phytanic acid	HMDB0000801	C20H40O2	312.3028	-	-1	
Propionic acid	HMDB0000237	C3H6O2	74.0368	-	-2	
Propionylglycine	HMDB0000783	*C5H9NO3*	131.0582	-	-1	
Propylene glycol	HMDB0001881	C3H8O2	76.0524	-	-2	
S-Adenosylhomocysteine	HMDB0000939	C14H20N6O5S	384.1216	-	-1	
S-Adenosylmethionine	HMDB0001185	C15H23N6O5S	399.1451	-	-1	
Sebacic acid	HMDB0000792	C10H18O4	202.1205	-	-1	
Serotonin	HMDB0000259	C10H12N2O	176.0950	-	-1	
Stearoylcarnitine	HMDB0000848	C25H49NO4	427.3662	-	-1	
Suberic acid	HMDB0000893	*C8H14O4*	174.0892	-	-1	
Suberylglycine	HMDB0000953	C10H17NO5	231.1107	-	-1	
Succinic acid semialdehyde	HMDB0001259	*C4H6O3*	102.0317	-	-1	

Succinylacetoacetate	HMDB0240258	C8H10O6	202.0477	-	-2	
Succinylacetone	HMDB0000635	C7H10O4	158.0579	-	-1	
Taurocholic acid	HMDB0000036	C26H45NO7S	515.2917	-	-1	
Tetradecanoylcarnitine	HMDB0005066	C21H41NO4	371.3036	-	-1	
Tiglylglycine	HMDB0000959	*C7H11NO3*	157.0739	-	-1	
Ureidosuccinic acid	HMDB0000828	C5H8N2O5	176.0433	-	-1	
Valyl-Proline	HMDB0029135	C10H18N2O3	214.1317	-	-1	
Vanillylmandelic acid	HMDB0000291	C9H10O5	198.0528	-	-1	

Molecular formulas in italics indicate that there are other metabolites with the same formula (isomers) present in the panel. In case isomers could not be distinguished (either because they had the same retention time (RT) or the RT was not known), they are combined in a single row.

AADC = aromatic l-amino acid decarboxylase, DHP = dihydropyrimidinase, HMDB = Human Metabolome Database, IVA = isovaleric acidaemia, NANS = N-acetylneuraminic acid synthase, SSADH = succinic semialdehyde dehydrogenase.

a) Identification (ID) levels: -2 = no peaks with a corresponding *m/z* identified (no reference compound-based RT available), -1 = no peaks with both a corresponding *m/z* and RT identified, 1 = peak(s) with corresponding *m/z* and RT identified, 2 = peak(s) with only corresponding *m/z* identified (no reference compound-based RT available).

b) RT is based on measurements in an IEM patient with a known deviation in the metabolite instead of on a reference compound.

Supplementary Table 3. N per sex and age group of the 87 selected control samples.

n		Age (years)				Total
		0-2	2-5	5-10	10-15	
Sex	Male	19	10	8	11	48 (55%)
	Female	10	10	5	14	39 (45%)
Total		29 (33%)	20 (23%)	13 (15%)	25 (29%)	87 (100%)

Samples were selected based on the following criteria: (1) subject age 0-15 years old, (2) collected in 2016, 2017 or 2018, (3) erythrocyte and leukocyte count in CSF within reference range, (4) if available, routine CSF parameters hemoglobulin, bilirubin, IgG production, glucose, lactate and protein within reference range, and (5) clinical data of the subject, as investigated by a pediatric neurologist, did not suggest the presence of a disease associated with abnormal CSF results. The most important reasons for performing a lumbar puncture in the patients of this study population (together with a commonly suspected associated disease) were: the diagnostic work-up of fever (meningitis), epilepsy (glucose transporter type 1 deficiency syndrome (GLUT1DS)), movement disorders (dopa-responsive dystonia, GLUT1DS), leukemia with central nervous system localization, and increased intracranial pressure (ICP) with normal imaging results. In this latter case, CSF is expected to be normal but still routinely collected and analyzed. For each case included in the control cohort, the suspected neurological disorder was excluded.

Supplementary Table 4. Resulting p-values and q-values (= FDR-adjusted p-values) from a Wilcoxon signed-rank test comparing the intensities of the 20 metabolites with highest intensity and 10 metabolites used as biomarker in this study between males (n = 48) and females (n = 39).

	p-value	q-value
2-Hydroxy-3-methylbutyric acid	0.22	0.93
3-Hydroxyisovaleric acid	0.46	0.93
3-Methoxytyrosine	0.34	0.93
4-Hydroxybutyric acid	0.82	0.93
5-Hydroxyindoleacetic acid	0.98	0.98
5-Hydroxy-L-tryptophan	0.76	0.93
Betaine	0.76	0.93
Citric acid	0.45	0.93
Creatine	0.76	0.93
Creatinine	0.01	0.16
D-Galactose/D-Glucose	0.28	0.93
Dihydrothymine	0.86	0.93
Dihydrouracil	0.97	0.98
Erythronic acid	0.84	0.93
Homovanillic acid	0.53	0.93
Hypoxanthine	0.13	0.93
Isoleucine	0.79	0.93
Isovalerylcarnitine	0.83	0.93
L-Acetylcarnitine	0.64	0.93
L-Carnitine	0.39	0.93
Leucine	0.87	0.93
L-Glutamine	0.53	0.93
L-Methionine	0.44	0.93
L-Tryptophan	0.52	0.93
L-Tyrosine	0.58	0.93
L-Valine	0.64	0.93
N-acetylmannosamine	0.83	0.93
N-acetylneuraminic acid	0.40	0.93
Phenylalanine	0.63	0.93
Pyroglutamic acid	0.64	0.93

No significant differences were found.

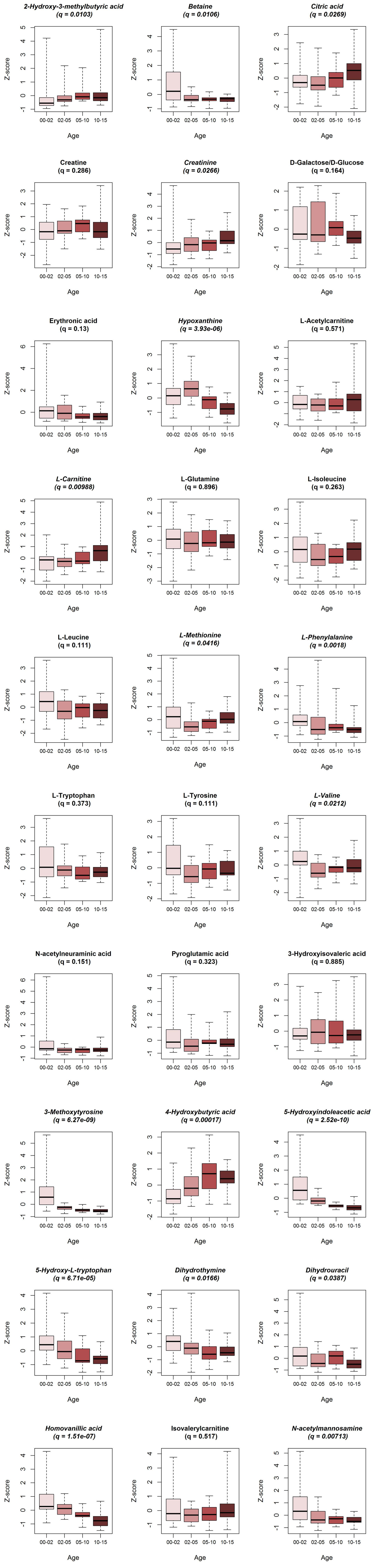
2-Hydroxy-3-methylbutyric acid (q = 0.0103)
Betaine (q = 0.0106)
Citric acid (q = 0.0269)
Creatine (q = 0.286)
Creatinine (q = 0.0266)
D-Galactose/D-Glucose (q = 0.164)
Erythronic acid (q = 0.13)
Hypoxanthine (q = 3.93e-06)
L-Acetylcarnitine (q = 0.571)
L-Carnitine (q = 0.00988)
L-Glutamine (q = 0.896)
L-Isoleucine (q = 0.263)
L-Leucine (q = 0.111)
L-Methionine (q = 0.0416)
L-Phenylalanine (q = 0.0018)
L-Tryptophan (q = 0.373)
L-Tyrosine (q = 0.111)
L-Valine (q = 0.0212)
N-acetylneuraminic acid (q = 0.151)
Pyroglutamic acid (q = 0.323)
3-Hydroxyisovaleric acid (q = 0.885)
3-Methoxytyrosine (q = 6.27e-09)
4-Hydroxybutyric acid (q = 0.00017)
5-Hydroxyindoleacetic acid (q = 2.52e-10)
5-Hydroxy-L-tryptophan (q = 6.71e-05)
Dihydrothymine (q = 0.0166)
Dihydrouracil (q = 0.0387)
Homovanillic acid (q = 1.51e-07)
Isovalerylcarnitine (q = 0.517)
N-acetylmannosamine (q = 0.00713)
Z-score
Age
00-02
02-05
05-10
10-15

Supplementary Figure 1. Boxplot of Z-scores per age group for 30 selected IEM metabolites.

The Z-scores have been calculated based on the total group of 87 controls. The q-values (= FDR-adjusted p-values) result from on a Kruskal-Wallis test for age group with $q<0.05$ indicating a significant difference between age groups overall (indicated by titles in italics).

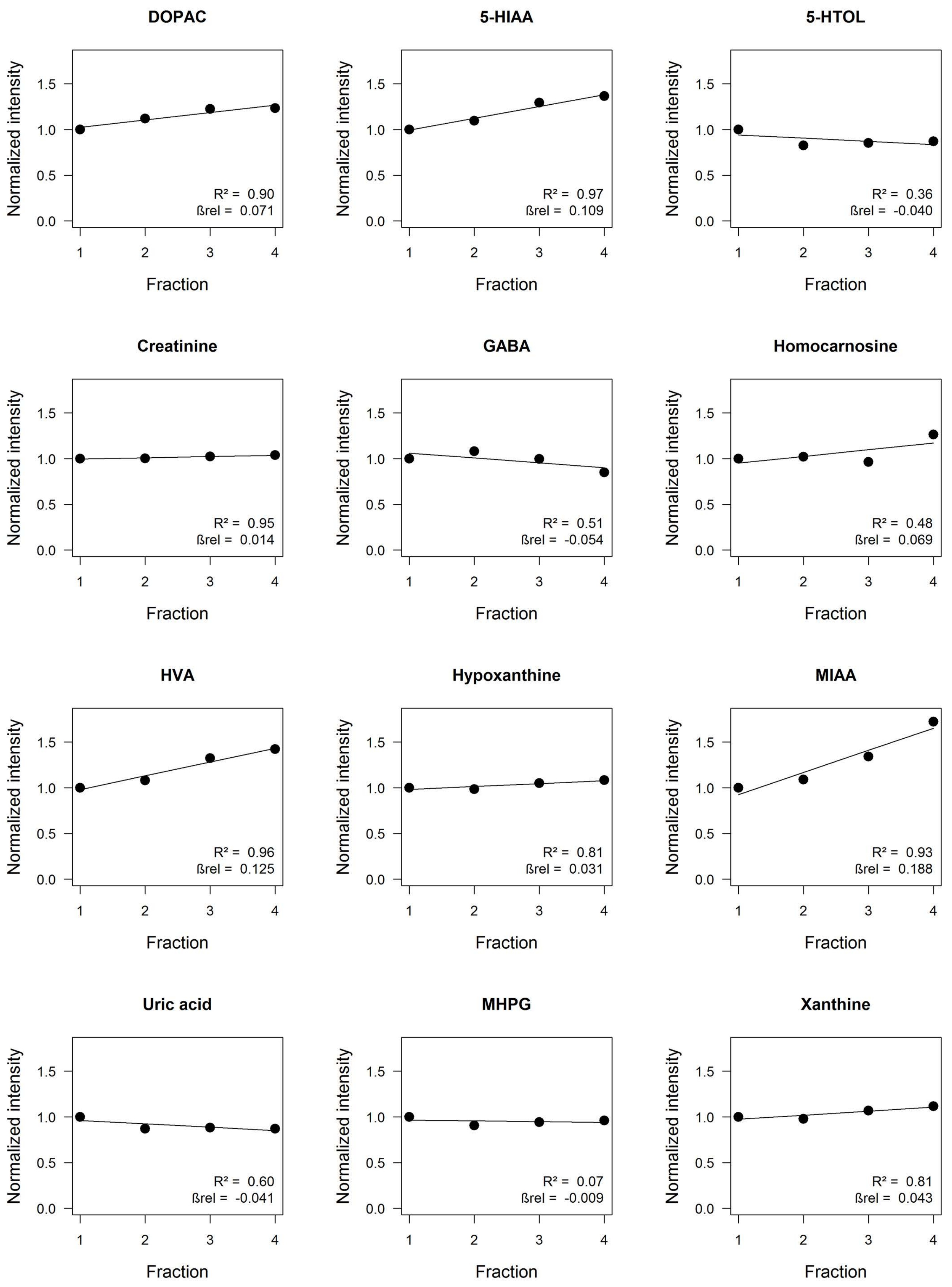
DOPAC
R² = 0.90
ßrel = 0.071
5-HIAA
R² = 0.97
ßrel = 0.109
5-HTOL
R² = 0.36
ßrel = -0.040
Creatinine
R² = 0.95
ßrel = 0.014
GABA
R² = 0.51
ßrel = -0.054
Homocarnosine
R² = 0.48
ßrel = 0.069
HVA
R² = 0.96
ßrel = 0.125
Hypoxanthine
R² = 0.81
ßrel = 0.031
MIAA
R² = 0.93
ßrel = 0.188
Uric acid
R² = 0.60
ßrel = -0.041
MHPG
R² = 0.07
ßrel = -0.009
Xanthine
R² = 0.81
ßrel = 0.043
Normalized intensity
Fraction
0.0
0.5
1.0
1.5
1
2
3
4

Supplementary Figure 2. Intensity plotted against fraction number for 12 metabolites that show a CSF concentration gradient according to literature.

Fractions: 1 = 0-5 ml, 2 = 5-10 ml, 3 = 10-15 ml, 4 = 15-20 ml. DOPAC = 3,4-Dihydroxyphenylacetic acid, 5-HIAA = 5-Hydroxyindoleacetic acid, 5-HTOL = 5-Hydroxytryptophol, GABA = gamma-Aminobutyric acid, HVA = Homovanllic acid, MIAA = Methylimidazoleacetic acid, MHPG = Vanylglycol.

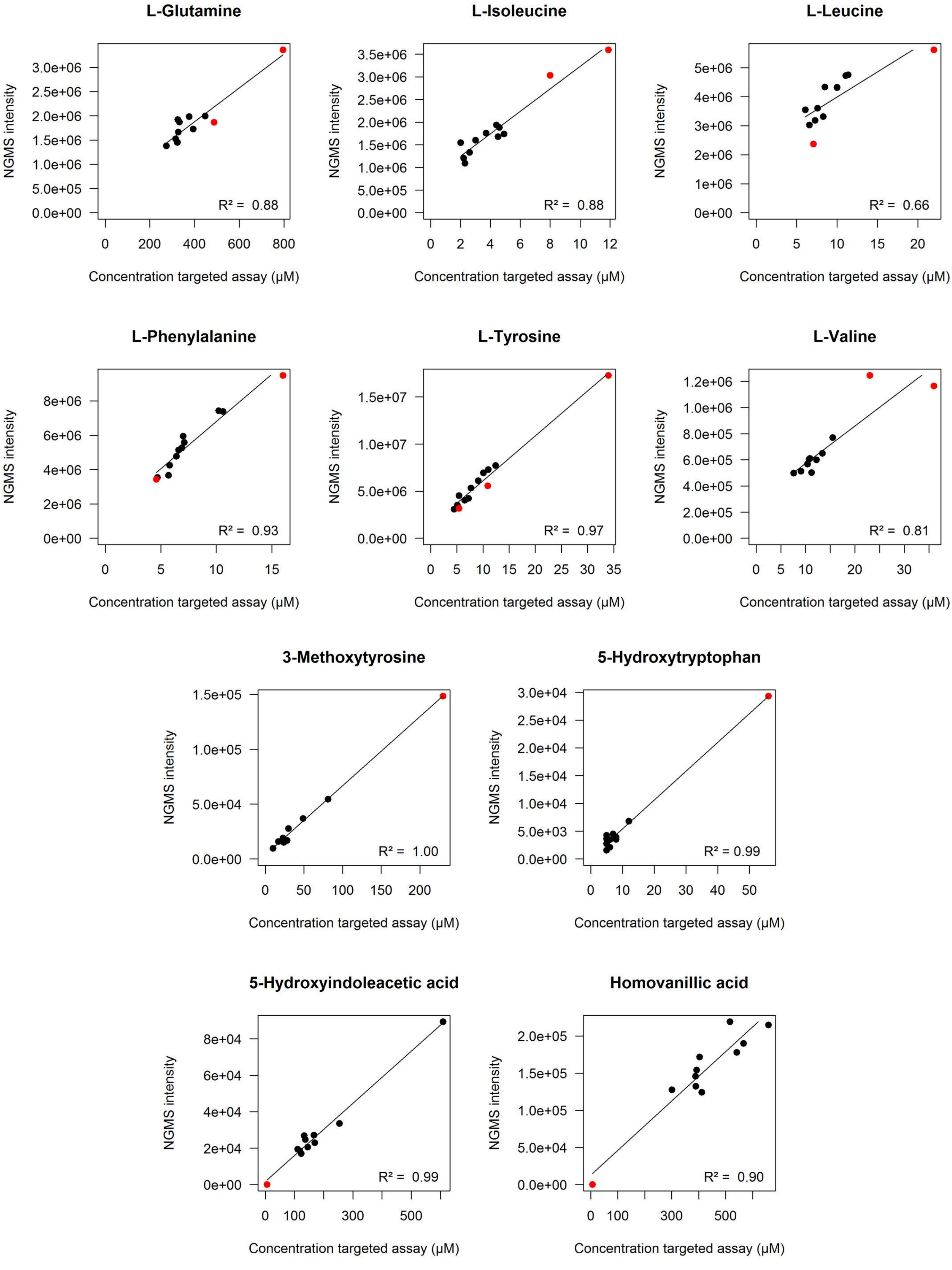
L-Glutamine
R² = 0.88
L-Isoleucine
R² = 0.88
L-Leucine
R² = 0.66
L-Phenylalanine
R² = 0.93
L-Tyrosine
R² = 0.97
L-Valine
R² = 0.81
3-Methoxytyrosine
R² = 1.00
5-Hydroxytryptophan
R² = 0.99
5-Hydroxyindoleacetic acid
R² = 0.99
Homovanillic acid
R² = 0.90
NGMS intensity
Concentration targeted assay (µM)

Supplementary Figure 3. Comparison of NGMS and targeted assays used in diagnostics for selected amino acids and neuro-transmitters.

Controls are indicated in black, patients in red. R^2 values and trend line were calculated by linear regression.
